# Supplementary material for: Proteome-Level Investigation of Vitis amurensis Calli Transformed with a Constitutively Active, Ca2+-Independent Form of the Arabidopsis AtCPK1 Gene
Source: Int J Mol Sci. 2023 Aug 24;24(17):13184. doi: 10.3390/ijms241713184 (PMC10487732; doi:10.3390/ijms241713184)
Supplement: Supplementary file 1 [file ijms-24-13184-s001.zip › Supplementary Material-R2.pdf]

Proteome-level investigation of *Vitis amurensis* calli transformed with a constitutively active, Ca^{2+}-independent form of the *Arabidopsis AtCPK1* gene

Galina N. Veremeichik[1], Dmitry V. Bulgakov[1], Yuliya A. Konnova[1], Evgenia V. Brodovskaya[1], Valeria P. Grigorchuk[1], Victor P. Bulgakov[1]

[1]Federal Scientific Centre of the East Asia Terrestrial Biodiversity of the Far East Branch of the Russian Academy of Sciences, Vladivostok, 690022, Russia

*Corresponding author

Address: Galina N. Veremeichik, Federal Scientific Centre of the East Asia Terrestrial Biodiversity FEB RAS, Vladivostok, 690022, Russia. E-mail: gala-vera@mail.ru,

Supplementary material

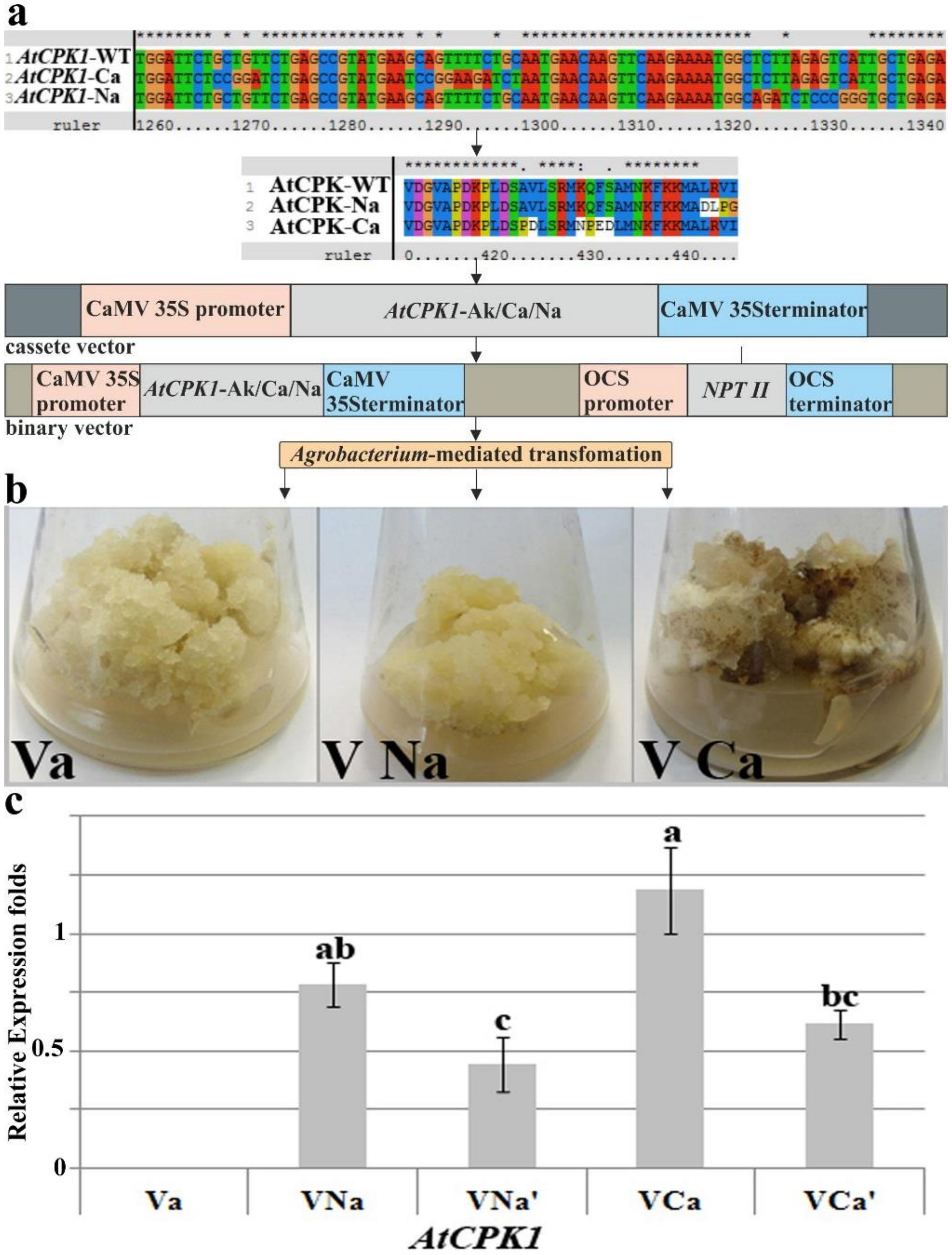

Figure S1. Schematic image (a) of the generation of genetic constructs carrying the native and mutant *AtCPK1* isoforms and activatory action of *AtCPK1* overexpression in plant cell cultures. The mutant forms of the *AtCPK1* gene (*AtCPK1-Na*, not active and *AtCPK1-Ca*, constitutively active) were engineered by Harper *et al*., (1994) and their activities were tested by Huang *et al*., (1996) and Harper *et al*., (1994), respectively. These forms were cloned into a cassette vector under regulation of the CaMV 35S promoter and terminator by Shkryl *et al*., (2011). The native form of *AtCPK1* was cloned from *A. thaliana* into cassette vectors under regulation of the CaMV 35S promoter and terminator by Shkryl *et al*., (2016). These constructs were transferred into binary vectors for *Agrobacterium*-mediated transformation of plant cells (Shkryl *et al*., 2011; Shkryl *et al*., 2016). The phenotypes of transgenic *Vitis amurensis* callus cultures (b) and *AtCPK1* gene expression (c). Data from real-time, quantitative PCR (mean ± standard error) represent measurements of three independent replicates from two different RNA isolations and are presented as relative expression levels normalized to the expression of the *V. vinifera* actin gene. Different letters above the bars indicate statistically significant differences of means ($P < 0.05$), Fisher's LSD. Va, untransformed callus line; V-Na and V-Na', callus lines transformed with nonactive *AtCPK1*, V-Ca and V-Ca', callus lines transformed with constitutively active *AtCPK1*. Cultures were grown for 4 weeks on WB/A medium (Veremeichik *et al*., 2017).

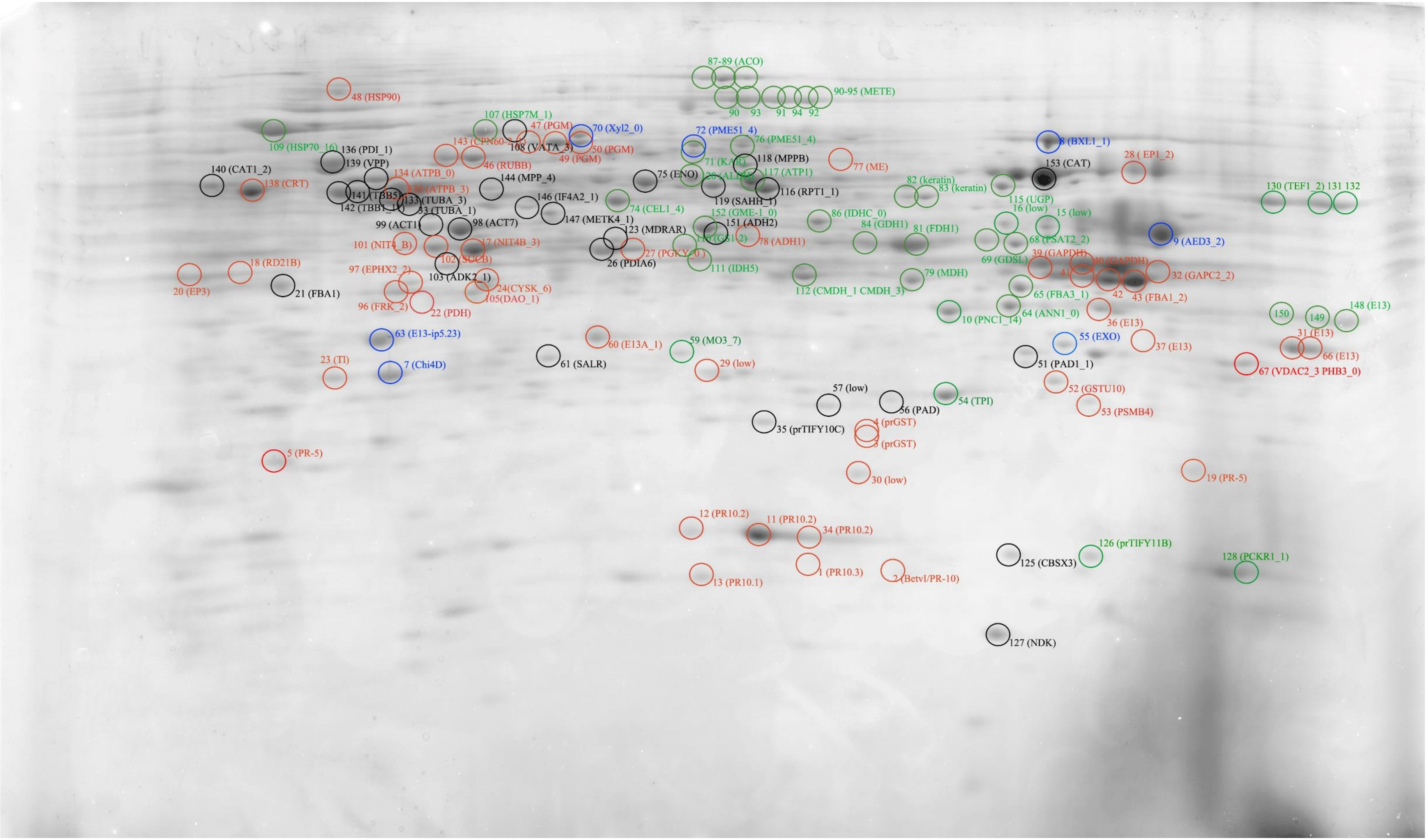

87-89 (ACO)
90-95 (METE)
90 93 91 94 92
48 (HSP90)
107 (HSP7M_1)
47 (PGM)
70 (Xyl2_0)
72 (PME51_4)
76 (PME51_4)
8 (BXL1_1)
109 (HSP70_16)
136 (PDI_1)
143 (CPN60-2_3)
108 (VATA_3)
50 (PGM)
49 (PGM)
71 (KAR)
118 (MPPB)
77 (ME)
28 (EP1_2)
139 (VPP)
46 (RUBB)
153 (CAT)
140 (CAT1_2)
134 (ATPB_0)
144 (MPP_4)
75 (ENO)
117 (ATP1)
82 (keratin)
138 (CRT)
135 (ATPB_3)
116 (RPT1_1)
83 (keratin)
130 (TEF1_2) 131 132
141 (TBB5)
133 (TUBA_3)
146 (IF4A2_1)
119 (SAHH_1)
115 (UGP)
142 (TBB1_1)
33 (TUBA_1)
74 (CEL1_4)
152 (GME-1_0)
86 (IDHC_0)
16 (low)
15 (low)
99 (ACT1)
98 (ACT7)
147 (METK4_1)
151 (ADH2)
84 (GDH1)
81 (FDH1)
123 (MDRAR)
9 (AED3_2)
101 (NIT4_B)
17 (NIT4B_3)
110 (GSL2)
78 (ADH1)
68 (PSAT2_2)
27 (PGKY_0)
69 (GDSL)
39 (GAPDH)
102 (SUCB)
26 (PDIA6)
40 (GAPDH)
18 (RD21B)
111 (IDH5)
97 (EPHX2_2)
79 (MDH)
41
32 (GAPC2_2)
103 (ADK2_1)
20 (EP3)
21 (FBA1)
24(CYSK_6)
112 (CMDH_1 CMDH_3)
65 (FBA3_1)
42
43 (FBA1_2)
105(DAO_1)
96 (FRK_2)
22 (PDH)
148 (E13)
10 (PNC1_14)
64 (ANN1_0)
150
149
36 (E13)
63 (E13-ip5.23)
55 (EXO)
31 (E13)
60 (E13A_1)
59 (MO3_7)
37 (E13)
66 (E13)
23 (TI)
61 (SALR)
51 (PAD1_1)
7 (Chi4D)
29 (low)
67 (VDAC2_3 PHB3_0)
52 (GSTU10)
57 (low)
54 (TPI)
56 (PAD)
53 (PSMB4)
35 (prTIFY10C)
4 (prGST)
3 (prGST)
5 (PR-5)
30 (low)
19 (PR-5)
12 (PR10.2)
11 (PR10.2)
34 (PR10.2)
126 (prTIFY11B)
125 (CBSX3)
128 (PCKR1_1)
13 (PR10.1)
1 (PR10.3)
2 (BetvI/PR-10)
127 (NDK)

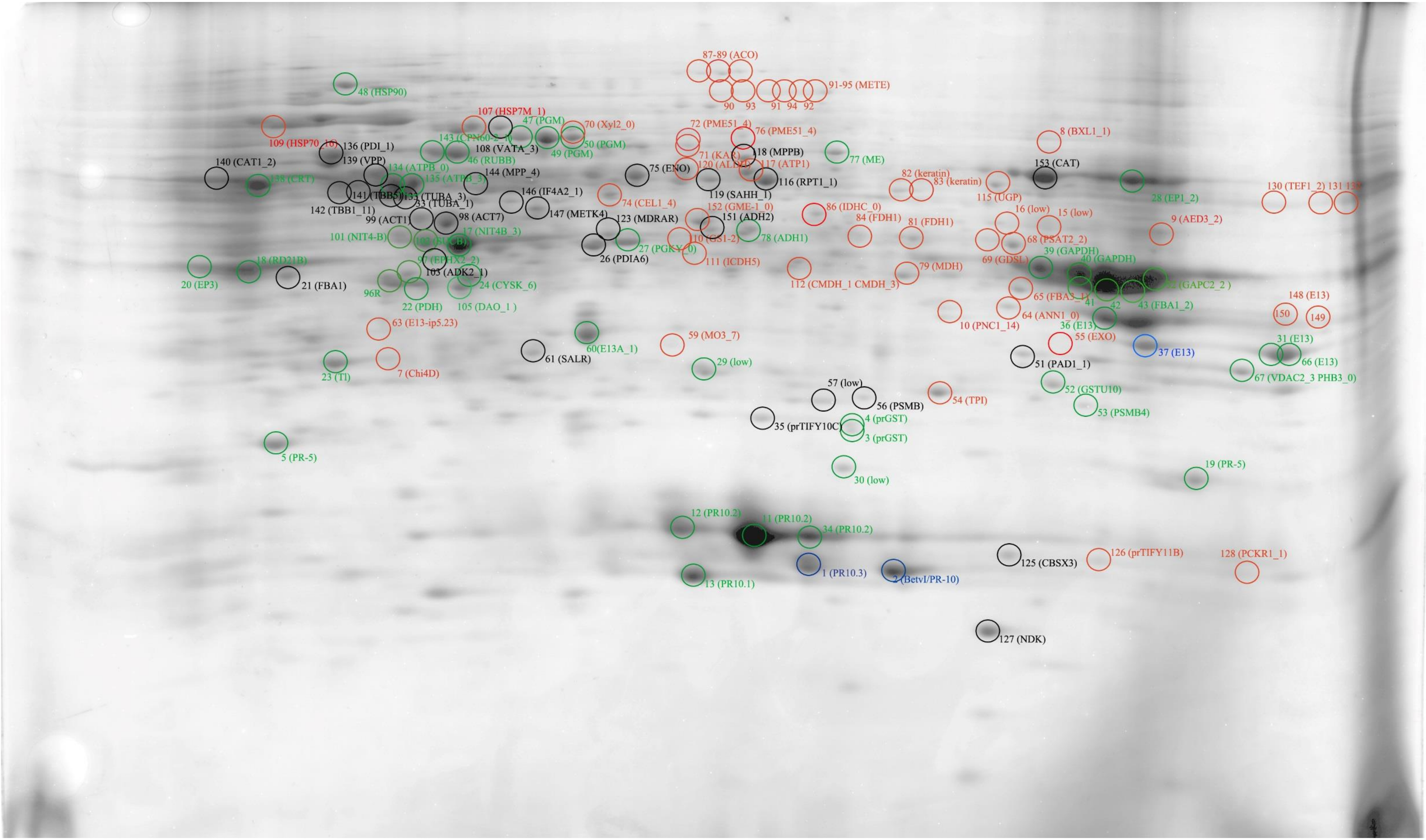

Figure S2. Representative Coomassie Brilliant Blue stained 2D gels of Va (top) and VaCa (bottom) calli.
